# Supplementary material for: Characteristics of creative individuals: An umbrella review protocol
Source: PLoS One. 2024 Oct 10;19(10):e0311851. doi: 10.1371/journal.pone.0311851 (PMC11469608; doi:10.1371/journal.pone.0311851)
Supplement: S2 File — (DOCX) [file pone.0311851.s002.docx]

*S2 File: Search Strategy for PsycINFO*

APA PsycInfo <1806 to July Week 3 2024>

1 creativity/ or divergent thinking/ 31180

2 (creativity or creative or divergent thinking).ti,ab. 65026

3 1 or 2 69307

4 (systematic review or meta-analysis or metanalysis or meta-analyses or metaanalyses or review).mp. 485410

5 "systematic review"/ or "literature review"/ or meta analysis/ 29006

6 4 or 5 485410

7 3 and 6 6308

8 (correlat* or relationship* or associat* or Pearson* or Cohen* or effect size or predictor* or moderator* or effect sizes).mp. 2065744

9 exp Statistical Correlation/ 11267

10 exp Statistical Estimation/ 9737

11 8 or 9 or 10 2074307

12 7 and 11 1873
